# Semen Cuscutae Administration Improves Hepatic Lipid Metabolism and Adiposity in High Fat Diet-Induced Obese Mice

**DOI:** 10.3390/nu11123035

**Published:** 2019-12-12

**Authors:** Jiyoung Moon, Min Jin Ha, Min-Jeong Shin, Oh Yoen Kim, Eun Hye Yoo, Juhyun Song, Ji Hyung Chung

**Affiliations:** 1 Department of Public Health Sciences, BK21PLUS Program in Embodiment: Health-Society Interaction, Graduate School, Korea University, Seoul 02841, Korea; answldud8503@naver.com (J.M.); 90minjin@hanmail.net (M.J.H.); mjshin@korea.ac.kr (M.-J.S.); dmsp1539@naver.com (E.H.Y.); 2 Department of Pharmaceutics and Pharmaceutical Chemistry, University of Utah, Salt Lake City, UT 84112, USA; 3 Department of Food Science and Nutrition, Dong-A University, Busan 49315, Korea; oykim@dau.ac.kr; 4 Department of Anatomy, Chonnam National University Medical School, Gwangju 61469, Korea; 5 Department of Biotechnology, College of Life Science, CHA University, Gyeonggi-do 11160, Korea

**Keywords:** Semen cuscutae (SC), arginase inhibitor, nitric oxide (NO), hepatic steatosis, obesity, peroxisome proliferator-activated receptor (PPAR)

## Abstract

Since arginase has been shown to compete with nitric oxide (NO) synthase, emerging evidence has reported that arginase inhibition improves obesity by increasing NO production. Semen cuscutae (SC), which is a well-known Chinese medicine, has multiple biological functions such as anti-oxidant function and immune regulation. In this study, we investigated whether the SC as a natural arginase inhibitor influences hepatic lipid abnormalities and whole-body adiposity in high-fat diet (HFD)-induced obese mice. The lipid accumulation was significantly reduced by SC treatment in oleic acid-induced hepatic steatosis in vitro. Additionally, SC supplementation substantially lowered HFD-induced increases in arginase activity and weights of liver and visceral fat tissue, while increasing hepatic NO. Furthermore, elevated mRNA expressions of sterol regulatory element-binding transcription factor 1 (SREBP-1c), fatty-acid synthase (FAS), peroxisome proliferator-activated receptor-gamma (PPAR-γ)1, and PPAR-γ2 in HFD-fed mice were significantly attenuated by SC supplementation. Taken together, SC, as a novel natural arginase inhibitor, showed anti-obesity properties by modulating hepatic arginase and NO production and metabolic pathways related to hepatic triglyceride (TG) metabolism.

## 1. Introduction

Arginase, which catalyzes the hydrolysis of l-arginine to urea, has been implicated in various diseases, such as obesity-associated vascular and metabolic dysfunction [1,2]. It has been known that arginase participates in the regulation of nitric oxide (NO) synthesis by competitively using an enzyme substrate for the three NOS isoforms, such as neuronal NOS (nNOS), inducible NOS (iNOS), and endothelial NOS (eNOS), through which arginase influences NO production [3,4]. Previous studies reported that increased arginase activity was found in obese individuals and those who have obesity related metabolic disorders, such as metabolic syndrome and endothelial dysfunction [5,6,7]. Therefore, arginase is involved in the reduction of NO production, so the blockage of its activity may improve endothelial dysfunction [2,8,9,10]. In addition, recent studies have shown that deletion of arginase 2 ameliorates high-fat induced obesity and the associated nonalcoholic fatty liver disease (NAFLD) [11,12]. In line with this, it was assumed that upregulated arginase expression in an obese condition is associated with the reduction in NO production, and thus with abnormal hepatic triglyceride (TG) metabolism and relevant metabolic pathways. Therefore, it is crucial to describe the beneficial effects of arginase suppression in NAFLD and the underlying mechanisms associated with obesity.

Based on previous evidence, the inhibition of arginase may be a critical issue to improve obesity-related pathologies, such as hepatic steatosis, in obese people [5,7,13,14]. Recently, there have been many attempts to find therapeutic natural compounds to control the hepatic lipid abnormality in obesity, because natural compounds might have fewer side effects than do synthetic drugs [15,16,17,18]. Semen cuscutae (SC) is an annual voluble parasitic herb belonging to the family Convolvulacea, and many biological functions of SC, for example, regulation of the endocrine system and immune system [19,20,21] and anti-oxidant [22,23,24] and anti-tumor functions [25], have been studied. Moreover, some findings have demonstrated protective effects of SC on liver injury [22,26,27]. However, the effects of SC on obesity and related hepatic metabolic abnormalities have not been investigated.

Therefore, we aimed to investigate the effects of SC on hepatic lipid abnormality in the high-fat diet (HFD)-induced obese mouse model, thereby suggesting the therapeutic potential of SC in obese-induced hepatic steatosis.

## 2. Materials and Methods

### 2.1. Arginase Activity Assay by Enzymatic Method

Sample powder (40 g) of Semen cuscutae purchased from the Kyungdong Traditional Medicine Store (Chungju-si, Chungbuk, Korea) was soaked with 95% ethanol (1 L) in a specific evaporating flask at 60 °C for 12 h using a rotary evaporator. This extraction was conducted three times and filtered using Millipore 0.45 μm filtration. The extract was then adjusted to a final volume of 25 mL. Arginase activity was enzymatically measured according to instructions from the Quantichrom arginase assay kit (Bioassay systems, Hayward, CA, USA). In brief, the livers of mice fed a normal diet were homogenized with cold radioimmunoprecipitation assay (RIPA) lysis buffer (Amresco; Solon, OH, USA) (0.025 g liver/500 μL lysis buffer) containing protease inhibitors (Roche Diagnostics; Mannheim, Germany) and centrifuged for 20 min at 4 °C. Upper supernatants were transferred into new 1.5 mL tubes and arginase activity was measured with this lysate from the liver. Arginine buffers were preheated at 37 °C. We made a 5× substrate buffer by mixing 4 vol of arginine buffer and 1 vol of Mn solution. We made 1 mM urea standard by mixing 24 μL of 50 mg urea/dL and 176 μL water. We added 50 uL of 1 mM urea standard and 50 uL water to each well of a 96-well plate. We added 40 μL of liver lysate to 3 separate wells (sample, positive control, or sample blank control well) of the 96-well plate. Then, 10 μL of 5× substrate buffer was added into the sample and positive control wells. In this study, we used nor-NOHA as a positive control, and arginase inhibitor to compare with SC. We added 10 μL of 5× substrate buffer into the sample and positive control wells. The sample blank control well was left without 5× substrate buffer and incubated for 2 h at 37 °C. Urea reagent was prepared by combining equal volumes of reagents A and B. We added 200 μL of urea reagent to all wells, and then 10 μL 5× substrate buffer was added to the sample blank control well. Then the plate was tapped to mix, incubated for 60 min at room temperature (RT), and read by optical density at 430 nm.

### 2.2. Cell Culture and Oleic Acid Induction for Steatosis

We obtained the HepG2 cells from American Type Culture Collection (ATCC, Rockville, MD, USA) and grew them in DMEM (high-glucose Dulbecco’s modified Eagle medium; Gibco, Gaithersburg, MD, USA) supplemented with 10% heat-inactivated FBS (fetal bovine serum) containing 100,000 Units/L penicillamine and 100 mg/L streptomycin obtained from Gibco. The cell cultures were maintained at 37 °C in a humidified atmosphere of 95% air and 5% CO_2_, and the medium of the cells was changed every two days. To produce the steatosis, the HepG2 cells were treated with 1.5 mM OLA (oleic acid; Sigma-Aldrich; St. Louis, MO, USA) or ethanol as a control for 24 h. For the cell viability assay, human hepatoma HepG2 cells (1 × 105 cells) were serum-starved overnight and treated with various concentrations of SC (0, 0.5, 1, 5, 10 μg/mL) for 24 h. We treated control cells with DMSO and measured cell viability by using 1 g/mL (3-(4,5-dimethylthiazol-2-yl)-2,5-diphenyltetrazolium bromide, MTT), followed by the addition of the MTT solution to each well, after which we incubated it at 37 °C for 1 h. After incubation, we measured absorbance at a wavelength 570 nm with a NanoQuant microplate reader (Tecan Trading AG, Maennedorf, Switzerland).

### 2.3. Lipid Content by SC Treatment

The seeded hepG2 cells (2 × 105 cells) were serum-starved overnight in 48-well culture plates and then treated with 1.5 mM OLA and SC (0, 0.5, 1, 5, 10 μg/mL) for 24 h. The treated cells were washed with PBS (phosphate-buffered saline) and fixed with 10% formalin for 1 h at RT, followed by washing of the cells with 60% isopropanol. After that, the cells were stained with Oil Red O for 10 min at RT and washed 4 times with distilled water. Images for each dish were captured using a microscope (Olympus Corporation, Tokyo, Japan). We added 3.6 mL of 100% isopropanol to the cells for 10 min and measured absorbance at 500 nm using a spectrophotometer (PerkinElmer, Waltham, MA, USA). AdipoRed assay (Lonza, Walkersville, MD, USA) was used to quantify the TG content according to the manufacturer’s protocol. We added 1.5 mM OLA and various concentrations of SC to the cells (2 × 105 cells) in 48-well plates and washed the cells with PBS. The cells were treated with 140 μL AdipoRed in 5 mL PBS for 15 min at RT. Absorbance was measured with a spectrophotometer (PerkinElmer, Waltham, MA, USA) at 485 nm and 535 nm.

### 2.4. High-Fat Diet Induced Obese Animals and Study Design

We purchased four-week-old male C57BL/6 mice (*n* = 26) from DBL (Daehan-BioLink Co., Chungbuk, Korea) for use in this experiment. After adaptation for 1 week, the animals were randomly assigned into 3 groups: control (CTL, *n* = 9), high-fat diet (HFD; 40% fat for total energy, *n* = 7), and SC (HFD with SC, *n* = 10) groups for 12 weeks. The normal diet was based on the AIN-76 rodent diet. The HFD was the same as the normal diet, except that it contained 200 g fat/kg (170 g lard plus 30 g corn oil) and 1% cholesterol (Table 1). They were housed in a pathogen-free environment with a controlled temperature (18–24 °C) and humidity (50%–60%). We routinely recorded daily food intake and weekly body weight throughout the experimental period. This procedure was approved by the Institutional Animal Care and Use Committee as governed by the National Institute of Health’s “Guide for the Care and Use of Laboratory Animals” and by the Committee on Animal Experimentation and Ethics of Korea University (KUIACUC-2015-150).

### 2.5. Sample Collection of Animals

At the end of the 12-week experiment period, the mice were sacrificed by anesthetizing with 30 mg kg^−1^ Zoletile (Vibrac, Carros, France) mixed with 10 mg kg^−1^ Rompun (Bayer Korea; Seoul, Korea) after a 12-h fast. We collected the blood samples from the abdominal inferior vena cava and heart, then transferred them to vacutainer tubes for serum. The blood samples were centrifuged at 1390 × *g* for 15 min at 4 °C and stored at −80 °C until further use. Liver and four visceral white adipose tissues (WAT: epididymal, perirenal, retroperitoneal, and mesenteric fat) were extracted, washed with 1× PBS, weighed (in g), rapidly frozen using liquid nitrogen, and stored in the freezer at −80 °C.

### 2.6. Measurement of Blood Biochemical Parameters

We measured serum concentrations of total cholesterol (TC), triglyceride (TG), and glucose enzymatically using commercial kits (Asan Pharmaceutical; Seoul, Korea).

### 2.7. Analysis of Nitric Oxide (NO) and Arginase Activity of the Liver

We measured NO from the liver tissue and serum using a Nitrate/Nitrite Colorimetric Assay kit (Cayman Chemical; Ann Arbor, MI, USA). Arginase activity with the cell and liver tissue lysates was measured using Quantichrome arginase assay kit (BioAssay Systems, Hayward, CA, USA). The samples lysed in cold buffer mixed with 50 mM Tris-HCl with pH 7.5, 0.1 mM EDTA, and protease inhibitors were centrifuged for 20 min at 14,000 *g*.

### 2.8. Hepatic RNA Extraction and Semi-Quantitative RT-PCR

To evaluate the hepatic mRNA expression of several genes, total RNA was isolated from 50 mg liver using the QIAzolLysis reagent in the RNeasy Lipid Tissue Mini Kit (Qiagen, Valencia, CA, USA) according to the manufacturer’s protocol. cDNA was synthesized from 1 μg of RNA using oligo-dT and SuperscriptTM II reverse transcriptase (Invitrogen, Carlsbad, CA, USA). One microgram of the cDNA was amplified by quantitative real-time PCR using the SYBR Green PCR Kit (Qiagen, Hilden, Germany). PCR was conducted in QuantStudio™ 6 Flex Real-Time PCR System (Applied Biosystems, Foster City, CA, USA) and the conditions were as follows: 15 min at 95 °C, followed by 40 thermal cycles of 94 °C for 30 s, 60 °C for 20 s, and 72 °C for 30 s. Target-specific primers for real-time PCR were designed using the Primer Express^®^ software. The data that were obtained were analyzed using the comparative cycle threshold (Ct) method and were normalized by the GAPDH expression value. Melting curves were generated for each PCR reaction to ensure the purity of the amplification product. The delta–delta-cycle threshold (2^-∆∆Ct^) method was used to calculate changes in gene expression as a relative fold difference between the experimental and endogenous control samples.

### 2.9. Western Blot Analysis

To evaluate the hepatic protein expression of AMPK, the protein of the liver was extracted and pooled using homogenization in cold RIPA (radioimmunoprecipitation assay) buffer purchased from Amresco (Solon, OH, USA) with protease inhibitors from Roche Diagnostic (Mannheim, Germany) and phosphatase inhibitors from Sigma-Aldrich. After determining protein content by the bicinchoninic acid assay (Bio-rad, Richmond, CA, USA), equal amounts of protein lysates were mixed with 5× loading buffer (1 M Tris–HCl, pH 6.8, a trace amount of bromophenol blue, 50% glycerol, 10% SDS, and DW) and lysis buffer and denatured at 95 °C for 5 min. Samples were subjected to 10% sodium dodecyl sulfate-polyacrylamide gel. After electrophoresis, proteins were electrophoretically transferred from the gel onto PVDF membrane in buffer (2.5 mM Tris, 19.2 mM glycine pH 8.3) at 0.3 mA/cm^2^ for 30 min at RT. Residual binding sites on the membrane were blocked by incubating the membrane in TBS (pH7.6) containing 0.1% Tween 20 and 5% nonfat dry milk for 1 h at RT. The blots were washed in TBS containing 0.1% Tween 20 and then incubated with appropriate antibody overnight at 4 °C. After washing, the membrane was incubated with anti-rabbit or mouse IgG Ab conjugated with HRP, and bands were visualized using enhanced chemiluminescence purchased from Young In Frontier (Seoul, Korea) and quantified by densitometry using an Alphaview^®^ software (Cell Biosciences, Santa Clara, CA, USA). Western blot analysis was performed using specific antibodies for AMPKα and phospho-AMPKα (Thr172) (Cell Signaling Technology, Danvers, MA, USA).

### 2.10. Statistical Analysis

Statistical analysis was performed using Statistical Package for the Social Science (SPSS 21.0, SPSS Inc., Chicago, IL, USA). Results were represented as mean ± S.E. The differences among the experimental groups were analyzed using one-way analysis of variance (ANOVA) with Duncan’s multiple range analysis, and *p* < 0.05 was considered as the criterion of significance.

## 3. Results

### 3.1. Effect of SC on Lipid Accumulation in OLA-Induced Hepatic Steatosis In Vitro

**Figure 1:**
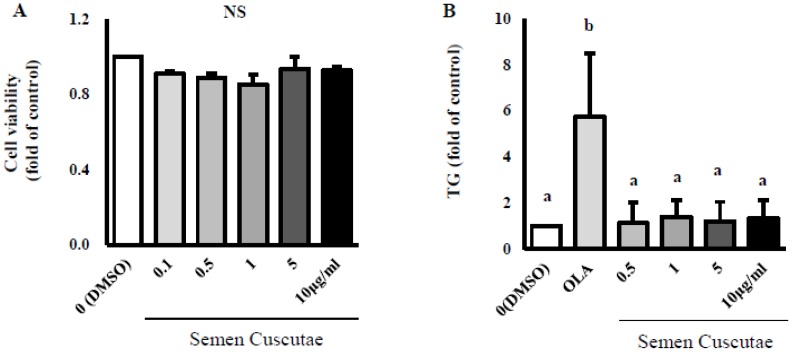

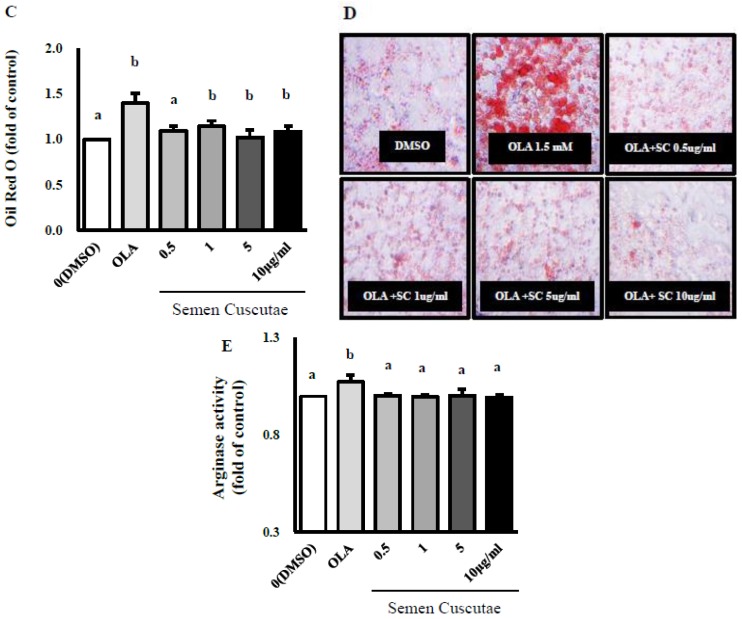
Effect of Semen cuscutae (SC) on lipid accumulation in oleic acid (OLA)-induced hepatic steatosis in vitro. Cell viability (**A**) triglyceride (TG) content (**B**), Oil Red O content (**C**), Oil Red O staining (**D**), and arginase activity (**E**) in OLA-induced hepatic steatosis in HepG2 cells. Cell viability was analyzed using the cells treated with different concentrations of Semen cuscutae for 24 h by MTT assay. TG content and intracellular lipid were measured by the AdipoRed assay and Oil Red O staining, respectively. Images were captured at 400× magnification. The data indicate the mean percentage levels compared with DMSO-treated cells. Values are expressed as means ± S.E.; *n* = 3. It is expressed in the same letter that there was no significant difference between the two groups (*p* < 0.05).

As shown in Figure 1A, SC treatment did not affect cell viability at any concentration (0.1–10 μg/mL). Figure 1B–D indicate that SC treatment at concentrations from 0.5 to 10 μg/mL attenuated lipid contents and intracellular TG content in OLA-treated HepG2 cells. To examine the effect of SC on arginase activity, we first enzymatically measured arginase activity in the liver of mice fed a normal diet (Graphic abstract). We found that the SC treatment dramatically reduced the arginase activity in a dose-dependent manner (121% at dose of 1 μg/mL, 100% at dose of 5 μg/mL, 72% at dose of 10 μg/mL of SC compared to the sample blank control (100%). Moreover, the level of arginase activity with 10 μg/mL of SC was lower than that of nor-NOHA (arginase inhibitor, 81% at dose of 10 μM) compared to that of CTL (Graphic abstract). In this study, SC treatment at all concentrations from 0.5 to 10 μg/mL significantly attenuated the increased arginase activity in 1.5 mM OLA-treated cells (Figure 1E).

### 3.2. SC Administration Ameliorates Diet-Induced Obesity

**Figure 2:**
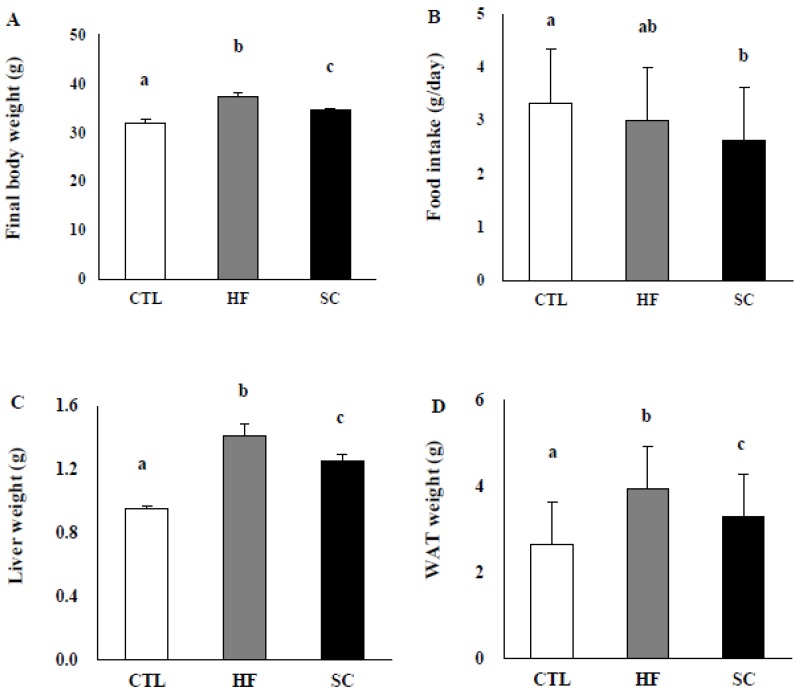
Effects of Semen cuscutae (SC) on diet-induced obesity and liver weight. (**A**) Final body weight, (**B**) food intake, (**C**) liver weight, and (**D**) white adipose tissue (WAT) weight in mice fed with normal (CTL) or high-fat diets (HFD). C57BL/6 mice were fed a normal diet or high-fat diet for 12 weeks. The mice in SC group were orally gavaged with 10 mg kg^−1^ Semen cuscutae dissolved in carboxymethylcellulose solutions. Daily food intake measurements and body weight were routinely recorded throughout the experimental period. Values are expressed as means ± S.E.; *n* = 3 mice per group. It is expressed in the same letter that there was no significant difference between the two groups (*p* < 0.05).

The body weight of HFD mice was significantly increased compared with that of CTL. The HFD group treated with SC showed a significant reduction of final body weight compared to the HFD group (Figure 2A), although there was no significant difference in total food intake between the HFD group and the SC group (Figure 2B). After 12 weeks, liver weights and WAT in the CTL and the SC groups were dramatically lower than those in the HFD group (Figure 2C,D).

### 3.3. Effects of SC on Hepatic and Circulating NO, and Circulating Biochemical Parameters

**Figure 3:**
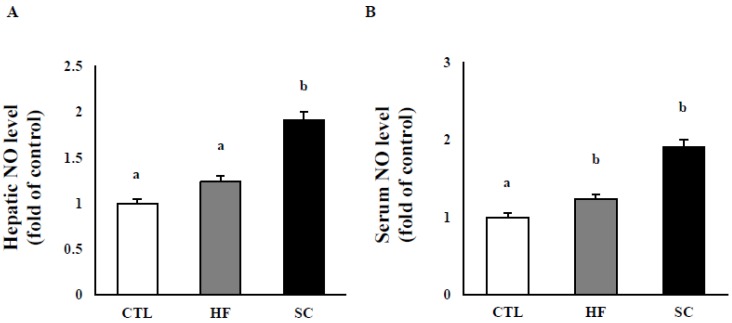
Effect of Semen cuscutae (SC) on levels of nitric oxide (NO) in diet-induced obesity. NO (nitrate + nitrite) in liver (**A**) and in serum (**B**) in mice fed with normal (CTL) or high-fat (HF) diets. Values are expressed as means ± SE; *n* = 3 mice per group. It is expressed in the same letter that there was no significant difference between the two groups (*p* < 0.05).

As shown in Figure 3A, the level of hepatic NO (nitrate + nitrite) in the SC group was significantly more elevated than was that of those in the CTL and the HF groups after the 12 week-intervention. Serum NO level was also increased by SC treatment after 12 weeks (Figure 3B). In addition, serum TG level was significantly decreased by SC treatment (33.7 ± 15.9 mg/dL) compared to the HF group (43.6 ± 3.7 mg/dL), but a significant difference was not observed between the SC group and the CTL (40.3 ± 2.2 mg/dL) group. The levels of serum TC in CTL (146 ± 10.6 mg/dL) and SC treatment groups (146.8 ± 6.4 mg/dL) were lower than those in HFD (164.8 ± 10.5), but there was no significance between the three groups. The glucose level in the SC treatment group (112.3 ± 4.7) was dramatically decreased compared to those in the CTL (165 ± 19.7) and HFD (161.8 ± 11.9) groups.

### 3.4. Effects of SC on Hepatic Lipid Metabolism In Vivo

**Figure 4:**
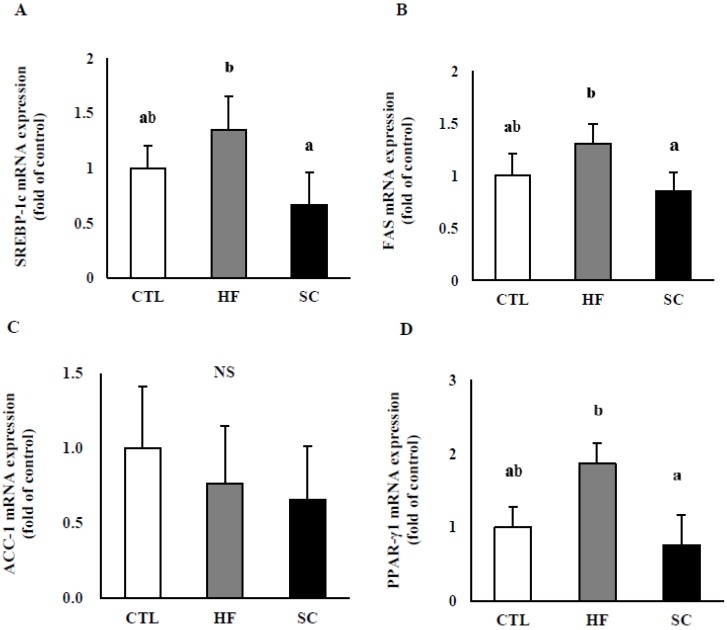

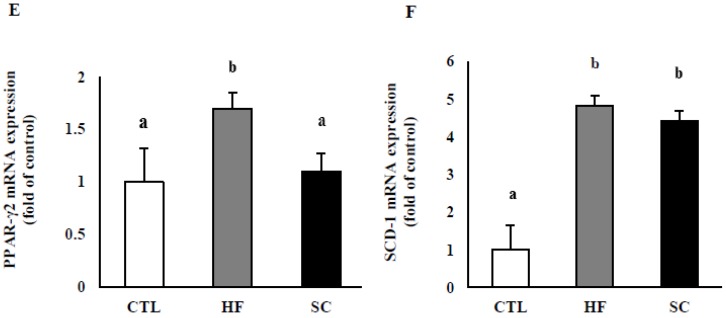
Effects of Semen cuscutae (SC) on the mRNA levels of several genes related to hepatic lipid metabolism in vivo. Total RNA extracted from each liver was subjected to real-time PCR analyses using primers specific for (**A**) sterol regulatory element-binding protein-1c (SREBP-1c), (**B**) fatty acid synthase (FAS), (**C**) acetyl-CoA carboxylase (ACC)-1, (**D**) peroxisome proliferator-activated receptor gamma (PPAR-γ) 1, (**E**) PPAR- γ 2, and (**F**) stearoyl-CoA desaturase-1 (SCD-1). Values are expressed as means ± S.E.; *n* = 3 mouse per group. It is expressed in the same letter that there was no significant difference between the two groups (*p* < 0.05).

To investigate the possible underlying mechanisms for SC’s effects, we evaluated the expression of several genes involved in hepatic TG metabolism (Figure 4A–F). The results demonstrated that SREBP-1c, FAS, PPAR-γ1, and PPAR-γ2 mRNA levels were significantly lower in the SC group compared with HF group. SCD-1 mRNA levels were significantly higher in both HF and SC groups than in the CTL group. On the other hand, the levels of ACC-1 mRNA were not significantly different between the three groups (CTL, HF, and SC).

### 3.5. Effects of SC on Phosphorylation of Hepatic AMPKα In Vivo

**Figure 5:**
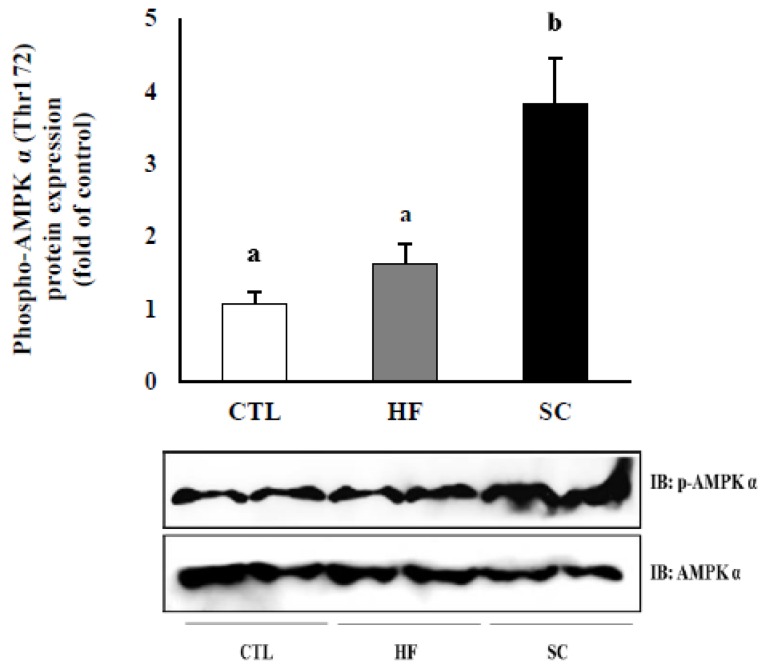
Effect of the SC on the activation of hepatic AMPKα in vivo. Protein levels in liver tissue with *p*-AMPKα and AMPKα levels showing a representative western blot image. The results are expressed as mean ± S.E. of mice, and the same letter indicates no significant difference between two groups (*p* < 0.05).

To investigate whether the pathway of phosphorylated AMPKα is necessary for hepatic lipid metabolism, which is ameliorated by SC, we evaluated the expression of phosphorylated AMPKα at Thr172 in the livers of three groups. There was no difference between the CTL and SC but the SC group had significantly increased phosphorylated AMPKα compared to the CTL and HF groups (Figure 5).

## 4. Discussion

SC, a natural compound collected from the dry ripe seeds of *Cuscuta chinensis* Lam, has been used as a natural medicine to control multiple biological responses [24,25,28,29]. In this study, we found that SC could attenuate hepatic lipid metabolism and whole-body adiposity by inhibiting arginase activity in diet-induced obese mice. Lipid accumulations measured by intracellular TG content and Oil Red O staining were also significantly reduced by SC treatment in OLA-induced hepatic steatosis in vitro. Based on our results, SC may contribute to the inhibition of lipid accumulation in obese people and to the improvement of hepatic metabolic abnormality.

NO, including nitrate and nitrite, contributes to glucose homeostasis by regulating microvascular blood flow, increasing insulin sensitivity and glucose uptake, and modulating inflammation and oxidative stress [30,31,32]. Therefore, insufficient production of NO may result in endothelial dysfunction and promote HFD-induced IR in obese status [31,32,33]. Our findings show that the hepatic NO level in HFD-fed mice supplemented with SC, as natural arginase inhibitor, was dramatically higher than those in HFD-only mice (HF group) and glucose levels in the SC group were significantly decreased compared to those in the CTL and HF groups. The possible mechanism may be further supported by our previous work and that of others [7,14,34,35,36,37,38]. Arginase inhibition promotes the expression of eNOS, which leads to the generation of NO in the HFD-fed mice livers and HepG2 cells [1,7,34]. It might be related to the activation of 59 adenosine monophosphate-activated protein kinase (p-AMPK) α, which is a member of the metabolic sensor protein kinase family and involved in the activation of eNOS regulating vascular endothelial function [35,36]. Besides, it has been reported that levels of vascular eNOS and AMPKα were reduced in HFD-fed rats [37], and the NO-AMPKα pathway may contribute to improving lipid disorders by inhibiting arginase activity in vitro and in vivo [14]. In this study, we clearly observed a significant increase in NO, AMPK activation, and a decrease of glucose by SC treatment. Considering our results and previous evidence, we suggest that SC might result in beneficial effects of arginase inhibition, thereby increasing the bioavailability of NO, improving the endothelial dysfunction, and regulating the glucose homeostasis in diet-induced obesity through AMPK signaling pathways [38,39,40].

It has been reported that inhibition of arginase reversed increased mRNA expressions of hepatic genes responsible for lipid metabolism [14]. In our study, increased mRNA levels of SREBP-1c, FAS, PPAR-γ1, and PPAR-γ2 in HFD-fed mice were significantly attenuated by SC administration. PPARs are expressed in many tissues, such as adipocytes, hepatocytes, and endothelial cells, and play a crucial role in lipid and glucose homeostasis. Especially, PPAR-γ is a transcription factor that implicates lipid production in the liver [41,42]. Recent studies reported that PPAR-γ is associated with the modulation of inflammation in fatty-liver disease and is involved in the development of hepatic steatosis by controlling fatty-acid transport, and a PPAR-γ agonist treatment prevents alcoholic liver injury in rats [43,44,45]. SREBP-1c, together with PPAR-γ is also a well-known gene associated with de novo lipogenesis and modulation of free fatty acid and TG levels [46]. It plays an important role in the transcriptional regulation of genes related to hepatic triglyceride synthesis and accumulation of lipid in the liver induced by an HFD [47,48,49]. SREBP-1c controls the expression of its downstream gene, such as FAS, which is involved in the synthesis of fatty acids and their incorporation into TG and phospholipids [50,51]. Therefore, increased SREBP-1c levels cause lipid accumulation in the liver and lead to hepatic steatosis [52,53,54,55,56,57]. Considering such evidence, inhibition of arginase activity by SC supplementation may regulate FAS by modulating SREBP-1c, thereby downregulating TG formations and the development of hepatic steatosis. Additionally, it was reported that the transcription of SREBP-1c and PPAR-γ was suppressed by the activated AMPK, and thus attenuated hepatic steatosis in HFD-induced animal models [58,59,60,61]. Our previous research reported that nor-NOHA, as an arginase inhibitor, significantly increased phosphorylated AMPK α, reduced the gene expression of ADRP and SCD-1 modulated by PPAR-γ2, and contributed to the development of fatty liver, suggesting the improvement of lipid metabolism in the liver and the anti-obesity effect in vivo and in vitro [14]. Given our results, SC may ameliorate the fat deposition and the development of hepatic steatosis by modulating SRBEP-1c and PPAR-γ by phosphorylated AMPK.

## 5. Conclusions

In conclusion, SC showed anti-obesity properties by modulating hepatic arginase activity, NO production, and metabolic pathways related to hepatic TG metabolism. Hence, we suggest the possibility that SC may be used as a natural medicine to treat hepatic lipid disorders and vascular dysfunction in obese people; however, further study is needed to confirm the possibility and these findings in the human obese model.

## Figures and Tables

**Table 1:** Composition of experimental diets (g/100 g diet).

Ingredients	CTL	HFD	HFD with SC
corn starch	15	15	15
casein	20	20	20
sucrose	50	34	34
corn oil	5	3	3
mineral mix ^1^	3.5	3.5	3.5
vitamin mix ^2^	1	1	1
cellulose	5	5	5
DL-methionine	0.3	0.3	0.3
choline bitartrate	0.2	0.2	0.2
lard	-	17	17
cholesterol	-	1	1
BHT	0.001	0.001	0.001
total	100	100	100

^1^ AIN-76 mineral mix. ^2^ AIN-76 vitamin mix. BHT, butylated hydroxytoluene; SC, Semen cuscutae; CTL, normal diet; HFD, high fat diet.

## Abbreviations

Nor-NOHA	N(omega)-hydroxy-nor-1-arginine
SC	Semen cuscutae
TG	triglyceride
TC	total cholesterol
WAT	white adipose tissue
SREBP-1c	sterol regulatory element-binding protein 1
FAS	fatty-acid synthase
PPAR-γ	peroxisome proliferator-activated receptor gamma
ADRP	adipose differentiation-related protein
eNOS	endothelial nitric oxide synthase
ACC-1	acetyl-coA carboxylase-1
SCD-1	stearoyl-CoA desaturase-1
p-AMPK	phosphorylated 5′ adenosine monophosphate-activated protein kinase

## References

[1] Romero M.J., Platt D.H., Tawfik H.E., Labazi M., El-Remessy A.B., Bartoli M., Caldwell R.B., Caldwell R.W. (2008). Diabetes-induced coronary vascular dysfunction involves increased arginase activity. Circ. Res..

[2] Peyton K.J., Liu X.M., Shebib A.R., Johnson F.K., Johnson R.A., Durante W. (2018). Arginase inhibition prevents the development of hypertension and improves insulin resistance in obese rats. Amino Acids.

[3] Sikka G., Pandey D., Bhuniya A.K., Steppan J., Armstrong D., Santhanam L., Nyhan D., Berkowitz D.E. (2013). Contribution of arginase activation to vascular dysfunction in cigarette smoking. Atherosclerosis.

[4] Pernow J., Jung C. (2013). Arginase as a potential target in the treatment of cardiovascular disease: Reversal of arginine steal?. Cardiovasc. Res..

[5] Hu H., Moon J., Chung J.H., Kim O.Y., Yu R., Shin M.J. (2015). Arginase inhibition ameliorates adipose tissue inflammation in mice with diet-induced obesity. Biochem. Biophys. Res. Commun..

[6] Kim O.Y., Lee S.M., Chung J.H., Do H.J., Moon J., Shin M.J. (2012). Arginase I and the very low-density lipoprotein receptor are associated with phenotypic biomarkers for obesity. Nutrition.

[7] Chung J.H., Moon J., Lee Y.S., Chung H.K., Lee S.M., Shin M.J. (2014). Arginase inhibition restores endothelial function in diet-induced obesity. Biochem. Biophys. Res. Commun..

[8] White A.R., Ryoo S., Li D., Champion H.C., Steppan J., Wang D., Nyhan D., Shoukas A.A., Hare J.M., Berkowitz D.E. (2006). Knockdown of arginase I restores NO signaling in the vasculature of old rats. Hypertension.

[9] Kovamees O., Shemyakin A., Pernow J. (2014). Effect of arginase inhibition on ischemia-reperfusion injury in patients with coronary artery disease with and without diabetes mellitus. PLoS ONE.

[10] Mahdi A., Pernow J., Kovamees O. (2019). Arginase inhibition improves endothelial function in an age-dependent manner in healthy elderly humans. Rejuvenation Res..

[11] Atawia R.T., Toque H.A., Meghil M.M., Benson T.W., Yiew N.H., Cutler C.W., Weintraub N.L., Caldwell R.B., Caldwell R.W. (2019). Role of Arginase 2 in Systemic Metabolic Activity and Adipose Tissue Fatty Acid Metabolism in Diet-Induced Obese Mice. Int. J. Mol. Sci..

[12] Liu C., Rajapakse A.G., Riedo E., Fellay B., Bernhard M.C., Montani J.P., Yang Z., Ming X.F. (2016). Targeting arginase-II protects mice from high-fat-diet-induced hepatic steatosis through suppression of macrophage inflammation. Sci. Rep..

[13] Moon J., Kim O.Y., Jo G., Shin M.J. (2017). Alterations in Circulating Amino Acid Metabolite Ratio Associated with Arginase Activity Are Potential Indicators of Metabolic Syndrome: The Korean Genome and Epidemiology Study. Nutrients.

[14] Moon J., Do H.J., Cho Y., Shin M.J. (2014). Arginase inhibition ameliorates hepatic metabolic abnormalities in obese mice. PLoS ONE.

[15] Thounaojam M.C., Nammi S., Jadeja R. (2016). Natural Products for the Treatment of Obesity, Metabolic Syndrome, and Type 2 Diabetes 2016. Evid. Based. Complement. Alternat. Med..

[16] Pan M.H., Lai C.S., Tsai M.L., Ho C.T. (2014). Chemoprevention of nonalcoholic fatty liver disease by dietary natural compounds. Mol. Nutr. Food Res..

[17] Ehsani N., Solouk A., Mardafkan N. (2018). Evaluation and Comparison of Synthetic and Natural Antioxidants Effectiveness. JBUMS.

[18] Zhang Y.Y., Zhang F., Thakur K., Ci A.T., Wang H., Zhang J.G., Wei Z.J. (2017). Effect of natural polyphenol on the oxidative stability of pecan oil. Food Chem. Toxicol..

[19] Lin M.K., Yu Y.L., Chen K.C., Chang W.T., Lee M.S., Yang M.J., Cheng H.C., Liu C.H., Chen D.C., Chu C.L. (2011). Kaempferol from Semen cuscutae attenuates the immune function of dendritic cells. Immunobiology.

[20] Liu X., Zeng Y.Q., Liang Y.Z., Zou C., Liu H., Qiu F., Liang C.L., Jin X.W., Su Z.R., Dai Z. (2016). Medicinal herbs Fructus corni and Semen cuscutae suppress allograft rejection via distinct immune mechanisms. Oncotarget.

[21] Pan H.J., Sun H.X., Pan Y.J. (2005). Adjuvant effect of ethanol extract of Semen Cuscutae on the immune responses to ovalbumin in mice. J. Ethnopharmacol..

[22] Yen F.L., Wu T.H., Lin L.T., Lin C.C. (2007). Hepatoprotective and antioxidant effects of Cuscuta chinensis against acetaminophen-induced hepatotoxicity in rats. J. Ethnopharmacol..

[23] Bao X., Wang Z., Fang J., Li X. (2002). Structural features of an immunostimulating and antioxidant acidic polysaccharide from the seeds of Cuscuta chinensis. Planta Med..

[24] Liu J.H., Ho S.C., Lai T.H., Liu T.H., Chi P.Y., Wu R.Y. (2003). Protective effects of Chinese herbs on D-galactose-induced oxidative damage. Methods Find Exp. Clin. Pharm..

[25] Nisa M., Akbar S., Tariq M., Hussain Z. (1986). Effect of Cuscuta chinensis water extract on 7, 12-dimethylbenz[a]anthracene-induced skin papillomas and carcinomas in mice. J. Ethnopharmacol..

[26] Kim E.Y., Kim E.K., Lee H.S., Sohn Y., Soh Y., Jung H.S., Sohn N.W. (2007). Protective effects of Cuscutae semen against dimethylnitrosamine-induced acute liver injury in Sprague-Dawley rats. Biol. Pharm Bull..

[27] Peng W.H., Chen Y.W., Lee M.S., Chang W.T., Tsai J.C., Lin Y.C., Lin M.K. (2016). Hepatoprotective Effect of Cuscuta campestris Yunck. Whole plant on carbon tetrachloride induced chronic liver injury in mice. Int. J. Mol. Sci..

[28] Yang L., Chen Q., Wang F., Zhang G. (2011). Antiosteoporotic compounds from seeds of Cuscuta chinensis. J. Ethnopharmacol..

[29] Yang H.M., Shin H.K., Kang Y.H., Kim J.K. (2009). Cuscuta chinensis extract promotes osteoblast differentiation and mineralization in human osteoblast-like MG-63 cells. J. Med Food..

[30] Lai Y.C., Gladwin M.T. (2016). Response by Lai and Gladwin to Letter Regarding Article, “SIRT3-AMP-Activated Protein Kinase Activation by Nitrite and Metformin Improves Hyperglycemia and Normalizes Pulmonary Hypertension Associated With Heart Failure With Preserved Ejection Fraction”. Circulation.

[31] Jiang H., Torregrossa A.C., Potts A., Pierini D., Aranke M., Garg H.K., Bryan N.S. (2014). Dietary nitrite improves insulin signaling through GLUT4 translocation. Free Radic. Biol. Med..

[32] Joost H.G., Tschop M.H. (2007). NO to obesity: Does nitric oxide regulate fat oxidation and insulin sensitivity?. Endocrinology.

[33] Sansbury B.E., Bhatnagar A., Hill B.G. (2014). Impact of nutrient excess and endothelial nitric oxide synthase on the plasma metabolite profile in mice. Front. Physiol..

[34] Ryoo S., Bhunia A., Chang F., Shoukas A., Berkowitz D.E., Romer L.H. (2011). OxLDL-dependent activation of arginase II is dependent on the LOX-1 receptor and downstream RhoA signaling. Atherosclerosis.

[35] Nguyen M.C., Park J.T., Jeon Y.G., Jeon B.H., Hoe K.L., Kim Y.M., Lim H.K., Ryoo S. (2016). Arginase Inhibition Restores Peroxynitrite-Induced Endothelial Dysfunction via L-Arginine-Dependent Endothelial Nitric Oxide Synthase Phosphorylation. Yonsei. Med. J..

[36] Demougeot C., Prigent-Tessier A., Marie C., Berthelot A. (2005). Arginase inhibition reduces endothelial dysfunction and blood pressure rising in spontaneously hypertensive rats. J. Hypertens.

[37] Bonnard C., Durand A., Vidal H., Rieusset J. (2008). Changes in adiponectin, itsreceptors and AMPK activity in tissues of diet-induced diabetic mice. Diabetes Metab..

[38] Forstermann U., Munzel T. (2006). Endothelial nitric oxide synthase in vascular disease: From marvel to menace. Circulation.

[39] O’Neill H.M. (2013). AMPK and exercise: Glucose uptake and insulin sensitivity. Diabetes Metab. J..

[40] Ruderman N.B., Carling D., Prentki M., Cacicedo J.M. (2013). AMPK, insulin resistance, and the metabolic syndrome. J. Clin. Invest..

[41] Grygiel-Gorniak B. (2014). Peroxisome proliferator-activated receptors and their ligands: Nutritional and clinical implications-a review. Nutr. J..

[42] Fajas L., Schoonjans K., Gelman L., Kim J.B., Najib J., Martin G., Fruchart J.C., Briggs M., Spiegelman B.M., Auwerx J. (1999). Regulation of peroxisome proliferator-activated receptor gamma expression by adipocyte differentiation and determination factor 1/sterol regulatory element binding protein 1: Implications for adipocyte differentiation and metabolism. Mol. Cell Biol..

[43] Gilardi F., Giudici M., Mitro N., Maschi O., Guerrini U., Rando G., Maggi A., Cermenati G., Laghezza A., Loiodice F. (2014). LT175 is a novel PPARalpha/gamma ligand with potent insulin-sensitizing effects and reduced adipogenic properties. J. Biol. Chem..

[44] Lee Y.J., Ko E.H., Kim J.E., Kim E., Lee H., Choi H., Yu J.H., Kim H.J., Seong J.K., Kim K.S. (2012). Nuclear receptor PPARgamma-regulated monoacylglycerol O-acyltransferase 1 (MGAT1) expression is responsible for the lipid accumulation in diet-induced hepatic steatosis. Proc. Natl. Acad. Sci. USA.

[45] Enomoto N., Takei Y., Hirose M., Konno A., Shibuya T., Matsuyama S., Suzuki S., Kitamura K.I., Sato N. (2003). Prevention of ethanol-induced liver injury in rats by an agonist of peroxisome proliferator-activated receptor-gamma, pioglitazone. J. Pharmacol. Exp. Ther..

[46] Ansari R.A., Husain K., Rizvi S.A. (2016). Role of Transcription Factors in Steatohepatitis and Hypertension after Ethanol: The Epicenter of Metabolism. Biomolecules.

[47] Zeng T., Xie K.Q. (2009). Ethanol and liver: Recent advances in the mechanisms of ethanol-induced hepatosteatosis. Arch. Toxicol..

[48] Bae J.S., Oh A.R., Lee H.J., Ahn Y.H., Cha J.Y. (2016). Hepatic Elovl6 gene expression is regulated by the synergistic action of ChREBP and SREBP-1c. Biochem. Biophys. Res. Commun..

[49] Horton J.D., Goldstein J.L., Brown M.S. (2002). SREBPs: Activators of the complete program of cholesterol and fatty acid synthesis in the liver. J. Clin. Invest..

[50] Ge C.X., Yu R., Xu M.X., Li P.Q., Fan C.Y., Li J.M., Kong L.D. (2016). Betaine prevented fructose-induced NAFLD by regulating LXRalpha/PPARalpha pathway and alleviating ER stress in rats. Eur. J. Pharmacol..

[51] Eberle D., Hegarty B., Bossard P., Ferre P., Foufelle F. (2004). SREBP transcription factors: Master regulators of lipid homeostasis. Biochimie.

[52] Liu H., Zhong H., Leng L., Jiang Z. (2017). Effects of soy isoflavone on hepatic steatosis in high fat-induced rats. J. Clin. Biochem. Nutr..

[53] Ferre P., Foufelle F. (2007). SREBP-1c transcription factor and lipid homeostasis: Clinical perspective. Horm. Res..

[54] Ferre P., Foufelle F. (2010). Hepatic steatosis: A role for de novo lipogenesis and the transcription factor SREBP-1c. Diabetes Obes. Metab..

[55] Shimomura I., Bashmakov Y., Horton J.D. (1999). Increased levels of nuclear SREBP-1c associated with fatty livers in two mouse models of diabetes mellitus. J. Biol. Chem..

[56] Koo S.H. (2013). Nonalcoholic fatty liver disease: Molecular mechanisms for the hepatic steatosis. Clin. Mol. Hepatol..

[57] Amemiya-Kudo M., Shimano H., Hasty A.H., Yahagi N., Yoshikawa T., Matsuzaka T., Okazaki H., Tamura Y., Iizuka Y., Ohashi K. (2002). Transcriptional activities of nuclear SREBP-1a, -1c, and -2 to different target promoters of lipogenic and cholesterogenic genes. J. Lipid Res..

[58] Li Y., Xu S., Mihaylova M.M., Zheng B., Hou X., Jiang B., Park O., Luo Z., Lefai E., Shyy J.Y. (2011). AMPK phosphorylates and inhibits SREBP activity to attenuate hepatic steatosis and atherosclerosis in diet-induced insulin-resistant mice. Cell Metab..

[59] Frederico M.J., Vitto M.F., Cesconetto P.A., Engelmann J., De Souza D.R., Luz G., Pinho R.A., Ropelle E.R., Cintra D.E., De Souza C.T. (2011). Short-term inhibition of SREBP-1c expression reverses diet-induced non-alcoholic fatty liver disease in mice. Scand. J. Gastroenterol..

[60] Sozio M.S., Lu C., Zeng Y., Liangpunsakul S., Crabb D.W. (2011). Activated AMPK Inhibits PPAR{alpha} and PPAR{gamma} Transcriptional Activity in Hepatoma Cells. Am. J. Physiol. Gastrointest. Liver Physiol..

[61] Villalpando-Arteaga E.V., Mendieta-Condado E., Esquivel-Solis H., Canales-Aguirre A.A., Galvez-Gastelum F.J., Mateos-Diaz J.C., Rodriguez-Gonzalez J.A., Marquez-Aguirre A.L. (2013). Hibiscus sabdariffa L. aqueous extract attenuates hepatic steatosis through down-regulation of PPAR-gamma and SREBP-1c in diet-induced obese mice. Food Funct..

